# Sex differences in perceptual responses to experimental pain before and after an experimental fatiguing arm task

**DOI:** 10.1186/s13293-019-0253-7

**Published:** 2019-08-07

**Authors:** Annamaria Otto, Kim Emery, Julie N. Côté

**Affiliations:** 1 Department of Kinesiology and Physical Education, McGill University, Montreal, Canada; 2 Feil & Oberfeld/CRIR Research Center, Jewish Rehabilitation Hospital, Laval, Canada

**Keywords:** Pressure pain, Sex, Repetitive work, Fatigue, Neck/shoulder, Questionnaires

## Abstract

**Background:**

The incidence and prevalence of musculoskeletal disorders (MSDs) is about twice as high in women compared to men, and those of the neck/shoulder region are particularly high among women. Fatigue and responses towards pain are known risk factor for MSDs. However, women have been shown to be less fatigable than men, but more sensitive to experimental pain. From a general standpoint, sex differences in the relationships between the fatigue and pain pathways are poorly understood. This may be due to differences in how men and women conceptually define the sensations of fatigue and pain. The objective of this study was to compare physical and verbal descriptors of fatigue and pain between men and women undergoing an experimental protocol where fatigue and pain were manipulated.

**Methods:**

Healthy adult volunteers (14 men and 14 women) underwent experimental pain tests to identify pressure pain threshold (PPT) at biceps brachii (BIC), anterior deltoid (AD), and upper trapezius (UT) followed by the Short-form McGill Pain Questionnaire (SF-MPQ) and Pain Catastrophizing Scale (PCS) before and after a repetitive arm task performed at shoulder height until reaching a rating of neck/shoulder perceived exertion, using the Borg Category Ratio 10 (CR10), greater than 8/10. PPT and MPQ data were analyzed using repeated measures analyses of variance (ANOVA) (time × sex). Correlational analyses were used to investigate relationships between pain measures with time and fatigue.

**Results:**

UT PPT was reduced following the fatiguing task (*p* ≤ 0.01). Men overall reported higher AD PPT levels compared to women (*p* ≤ 0.05). MPQ and PCS magnification scores were significantly higher after the fatiguing task (*p* ≤ 0.05), with no sex differences. Time to fatigue correlated with changes in AD PPT in men and with PCS scores in women.

**Conclusions:**

Findings suggest that mechanisms underlying the sensation of acute pain following a repetitive shoulder height task are closely linked with PPT changes in shoulder stabilizers (UT) irrespective of sex, and more so with physical pain responses in men and in attitudes towards pain in women. Sex differences in pain perception may contribute to a better understanding of sex-specific mechanisms underlying neck/shoulder MSDs.

## Background

Musculoskeletal disorders (MSDs) represent the leading cause of prolonged work disability worldwide [34]. Moreover, the prevalence of upper limb work-related MSDs (WMSDs) is unequally distributed among men and women, as women disproportionately report more neck/shoulder WMSDs than men [32]. The mechanisms underlying this difference in prevalence are poorly understood and may originate from factors related to either sex (biological and physiological traits) or gender (psychological and sociological characteristics). One mechanism that may contribute to this disparity is differences in acute pain pathways [5]. Studies have shown that women consistently report lower mechanical pressure pain thresholds (PPT) (i.e., the lowest amount of mechanical pressure applied on the skin over a muscle that elicits pain; not to be confused with pain tolerance, which would be the highest amount of pain that one can sustain), compared to men [1, 12, 27]. Particularly relevant to neck/shoulder WMSDs, women have been shown to report lower PPT than men in the upper trapezius, a main shoulder stabilizer that is actively engaged during work at shoulder height [13, 21]. Although studies have identified elements along the pain pathways, such as hormonal and brain structure and activation characteristics, that could help explain this sex difference (see [1] for review), the specific mechanisms underlying sex differences in acute mechanical pressure pain remain poorly understood.

Repetitive upper limb movements are a major risk factor for neck/shoulder WMSDs and may cause muscle fatigue and provoke fatigue-compensatory movement strategies [6, 23]. Following submaximal fatiguing isometric contractions, PPT in working muscles has primarily been shown to increase with time [19, 20]. However, this has mostly been found in women [17]. Furthermore, following submaximal dynamic contractions of the elbow flexors, women only reported higher PPT at the index finger [22]. However, few studies have investigated sex differences in PPTs related to neck/shoulder fatigue.

Psychosocial factors can also influence the response to experimental pain. Pain management techniques are proposed to act as pain-response mediators in experimental pain [9]. In response to a painful stimulus, women tend to engage in catastrophizing (i.e., exaggeration of the intensity of the pain experience) to a greater extent than men [1, 9]. In addition to pain catastrophizing, verbal descriptions of pain could provide important information regarding the pain experience. The Short-Form McGill Pain Questionnaire (SF-MPQ) provides a platform for people to report verbal descriptions of pain in the sensory and affective dimensions [24]. Very few studies have investigated the use of SF-MPQ following repeated or prolonged muscle contractions. Moreover, the SF-MPQ has not yet been used to compare verbal pain ratings of men and women following a dynamic work-like fatiguing task.

Therefore, the purpose of this study was to describe the sex-specific characteristics of the pain experience following neck/shoulder fatigue from shoulder height work. We hypothesized that pain measures would change with fatigue and that there would be sex differences in PPT and verbal descriptions of pain following a low-load, work-like task designed to fatigue the neck/shoulder musculature.

## Methods

### Design

A quasi-experimental study design with experimental pain tests and questionnaires administered before and after a neck/shoulder fatiguing task was used to compare pain responses between men and women in the pre- and post-fatigue conditions.

### Participants

A convenience sample of 29 healthy adults was solicited from the McGill University student population. Sample size was determined based on previous sample sizes and achieved power from similar research investigating sex differences in pressure pain threshold in rest and fatigue conditions. In resting conditions, women have been found to have statistically lower PPT with a sample size of *N* = 30 (15 men and 15 women) [3], and following fatiguing contractions, sex differences in PPT and pain ratings were detected in a sample size of *N* = 26 (13 women and 13 men) [14]. Participants were included if they were aged between 18 and 45 years. They were excluded if they had a history of being medically diagnosed with a neck/shoulder injury that required time away from work, any current acute or chronic pain conditions, neurological conditions that could affect one’s ability to perform the task, or use pain medication 24 h prior to the experimental session. Of the 29 volunteers, one participant was removed from the sample after indicating a score greater than “0” on a 10-point visual analog scale (VAS) for neck/shoulder pain upon arrival to the lab. Thus, the final group included 28 [14 men (age 22.93 (SD 1.82) and 14 women (age 23.36 (SD 2.84)] adult volunteers. All participants were right-hand dominant, generally healthy (assessed by the PAR-Q), and pain free (evaluated by the visual analog scale (VAS)). The experimental protocol took place at the Occupational Biomechanics and Ergonomics Laboratory at the Jewish Rehabilitation Hospital in Laval, Quebec, Canada. All participants provided written informed consent prior to participating in the protocol that was approved by the Research Ethics Board of the *Center for Interdisciplinary Research in Rehabilitation*.

### Experimental procedure

At the beginning of the experimental session, anthropometric measurements (height weight, skinfolds) were taken. A pressure pain test measurement procedure was then administered using a pressure algometer (Somedic AB, Farsta, Sweden, probe size of 1 cm^2^ surface area) applied at the bellies of the upper trapezius (UT), anterior deltoid (AD), and biceps brachii (BIC) muscle sites to identify PPT. Muscle sites were identified and marked prior to administering the pain tests to ensure that PPT was applied at the same place before and after the fatiguing task. In a seated position, the participant rested their right arm on a table with their shoulder flexed 90° and a straight elbow. Pressure was applied manually at a generally constant rate of 40 kPa/s [28]. To allow for return to baseline sensation but minimize fatigue recovery during the post-fatigue measurements [18], PPT trials were performed sequentially in the following order UT, AD, and lastly BIC, with 30-s rest in between each trial to the same muscle. This sequence was performed three times, resulting in three trials for each muscle. The participant was instructed to indicate when the sensation of pressure only changed to one of pressure and pain by pressing a button connected to the pressure algometer held in the opposite hand while keeping their eyes closed. With proper instructions, the pressure algometer technique to assess PPT has previously shown strong intra-observer [4], and strong test-retest reliability [16, 35].

After the PPT tests, participants completed the SF-MPQ and the Pain Catastrophizing Scale (PCS). The SF-MPQ contains 15 words that may reflect the experience of pain (e.g., “throbbing,” “aching”) in affective and sensory dimensions of pain. It uses a Likert scale with intensity rankings of none (0), mild (1), moderate (2), and severe (3) [24]. Participants were instructed to make a mark next to the score associated with each word to indicate the extent it represents their current pain experience. The Present Pain Intensity (PPI) score of the long-form McGill Pain Questionnaire and VAS were also included in the questionnaire to evaluate overall pain intensity. The VAS consisted of a horizontal line with a distance of 100 mm in length on which participants marked their perceptive pain intensity with anchors of no pain (0) to the worst possible pain (10). The PPI consisted of a 6-point choice between 0 (no pain) and 5 (excruciating), with participants checking the line next to the number that best reflected their current level of pain. The SF-MPQ is highly correlated with the long form of the McGill Pain Questionnaire, which is identified as valid and reliable in clinical samples [24]. The PCS is a self-report tool consisting of 13 items that measure the three dimensions of pain catastrophizing, which are magnification, rumination, and helplessness [31]. For each of the 13 items on the scale, there is a 5-point Likert scale from 0 (none) to 4 (all the time) that is used to indicate the degree of thought or feeling. The PCS has been shown to be both reliable and valid in assessing the three dimensions of pain catastrophizing in experimental samples [31]. Each participant received the same verbal instructions before completing the questionnaires to avoid potential researcher bias. For the SF-MPQ, they were instructed to refer to the pain they felt during the pressure pain test. For the PCS, they were instructed to “refer to any neck/shoulder pain that they themselves may have experienced in their lifetime.” This approach was chosen to assess any impact of the current protocol on general pain perceptions, since the PCS includes items that refer to individual attitudes towards pain in general, everyday contexts.

Subsequently, participants completed the experimental task designed to fatigue the muscles in the neck/shoulder area. The task consisted of a manual dexterity activity performed with the shoulder flexed 90° and the arm held horizontally at shoulder height. Participants were positioned an arm’s length away from the workstation. The workstation was vertical, individualized to shoulder height, and comprised of a board with washers and screws. For the task, participants were instructed to fasten six washers between 2 rows of 6 screws (12 total screws) and the depth of the washer (0.8 cm), with the screws spaced 0.4 cm apart. At the end of each minute during the task, participants were asked to verbally report their rating of perceived exertion (RPE) from the Borg CR-10 Scale [2]. The task was performed until reaching an RPE of 8/10 or the participant could no longer perform the task. Participants were unaware of these stoppage criteria. Immediately afterwards, the pain test was administered again to identify PPT on UT, AD, and BIC muscles. Subsequently, the SF-MPQ and PCS were completed with the same instructions as in the pre-fatigue condition.

### Data analysis

For PPT data, values were recorded in a chart and averaged to obtain per-subject and group mean values for the UT, AD, and BIC muscle sites in both the pre- and post-fatigue conditions. The SF-MPQ responses were evaluated based on total scores (0–45), sensory score (0–33), and affective score (0–12), as well as by using the mean VAS and PPI scores. The total score was summed from the responses for the 15 descriptive words, the sensory descriptor score was summed from the responses of items 1–11, and the affective descriptor score was summed from the responses of items 12–15 [24]. To score the VAS, the distance in millimeters from the left edge of the scale to the participant’s mark determined their score [7]. Completed PCS questionnaires were assessed based on the total score, as well as the three subscale scores evaluating rumination, helplessness, and magnification [30]. The total score was calculated by summing the responses of all 13 items, with a possible range of total scores from 0–52. Subscale scores were computed by summing the score of the responses for the following items: Rumination (items 8, 9, 10, 11), Helplessness (items 1, 2, 3, 4, 5, 12), and Magnification (items 6, 7, 13).

### Statistical analysis

An independent samples *t* test was run to assess sex differences on the time that it took for participants to reach the fatiguing task termination criteria (identified as time to Borg-8). Statistical tests were run to assess the effects of time (before and after the fatiguing task) and of sex on PPT values and questionnaire scores. The Shapiro-Wilk test was used to test the normality of data sets. For normally distributed data, two-way repeated measures ANOVA were used. The Friedman Test was used to analyze data that were not normal, while the Mann Whitney *U* test was used to compare between men and women.

Correlational analyses were run to assess the relationships between pain variables for the entire group and for men and women separately, using a merged then sex-stratified approach used previously that allows factors to be identified that could be either common or different between men and women [10]. For data that met normality assumptions, Pearson correlation coefficients were used, whereas for the data that did not meet assumptions of normality, Spearman’s rho correlations were used. Therefore, Pearson correlations were used to investigate the relationships between pre- and post-fatigue changes in PPT data and SF-MPQ, as well as between changes in PPT and time to Borg-8, whereas Spearman’s rho correlations were run between changes in PPT measures and changes in magnification scores of the PCS. Finally, Spearman’s rho correlations were run between pre-fatigue PCS measures and time to Borg-8. The interpretation of the strength of the correlations was adapted from Portney and Watkins [26] as follows: 0.00–0.25 = “Little or no relationship,” 0.26–0.50 = “Poor to Fair relationship,” 0.51–0.75 = “Moderate to Good relationship,” and > 0.75 = “Good to Excellent relationship.”

## Results

### Time to Borg-8

The average time participants performed the task until first reporting the RPE of 8 or higher was 6.35 ± 3.57 minutes. Independent samples *t* tests revealed that men (*M* = 5.42, SD = 3.11) and women (*M* = 7.29, SD = 3.87) did not differ in time to Borg-8 or higher during the neck/shoulder fatiguing task [*t*(26) = 1.41, *p* = 0.172].

### Pressure pain threshold

PPT data for all three muscle sites (UT, AD, and BIC) were found to be normally distributed according to the Shapiro-Wilk test of normality. Two-way repeated measures ANOVA (time × sex) showed some significant results for the UT and AD muscle sites (Table 1). Analysis of UT PPT data revealed a significant main time effect for the entire group; [*F*(1,26) = 15.96, *p* < 0.001]. UT PPT scores were lower in the post-fatigue condition compared to the pre-fatigue condition. For AD PPT data, there was a significant main sex effect [*F*(1,26) = 5.72, *p* = 0.024]. Men reported higher AD PPT levels than women, regardless of time. No other significant findings were observed for PPT data.

**Table 1:** PPT values at UT, AD, and BIC sites for the entire group, men and women. *P* values for the entire group represent the test between pre- and post-fatigue conditions and *p* values for men and women represent differences between the groups. Data are presented as mean ± standard deviation (*statistically significant at *p* < 0.05, **statistically significant at *p* < 0.01**)**

		Pre-fatigue	Post-fatigue	Significance
UT	Entire group	377.3 ± 126.90	326.27 ± 103.80**	*p* < 0.001
	Men	416.40 ± 123.43	359.19 ± 101.12	*p* = 0.086 (sex)
	Women	338.19 ± 122.14	293.36 ± 99.10	
AD	Entire group	304.42 ± 103.42	276.94 ± 99.86	*p* = 0.054
	Men	335.60 ± 101.30	324.90 ± 108.58	*p* = 0.024 (sex)
	Women	273.24 ± 99.29*	228.98 ± 62.96*	
BIC	Entire group	306.12 ± 102.22	285.40 ± 114.32	*p* = 0.121
	Men	342.38 ± 89.41	324.83 ± 129.74	*p* = 0.050 (sex)
	Women	269.86 ± 104.30	245.98 ± 83.43	

#### Questionnaires (Table 2)

**Table 2:** Average scores for pre- and post-fatigue SF-MPQ and PCS, as well as significant levels. *P* values represent the test between pre- and post-fatigue conditions for the entire group. Data are presented as mean ± standard deviation (*statistically significant at *p* < 0.05; **statistically significant at *p* < 0.01)

Short Form McGill Pain Questionnaire			
	Pre-fatigue	Post-fatigue	Significance
Total score (all 15 items)	5.29 ± 2.93	7.61 ± 6.01*	*p* = 0.026
Sensory scores (items 1–11)	4.93 ± 2.76	6.71 ± 4.71*	*p* = 0.031
VAS scores	1.61 ± 1.20	2.65 ± 1.46**	*p* = 0.002
Affective scores (items 12–15)	0.36 ± 0.68	0.85 ± 1.67	*p* = 0.13
PPI Scores	1.29 ± 0.71	1.61 ± 0.79	*p =* 0.052
Pain Catastrophizing Scale			
	Pre-Fatigue	Post-Fatigue	Significance
Total scores (all 13 items)	9.25 ± 8.12	9.71 ± 8.41	*p* = 0.13
Rumination scores (items 8, 9, 10, and 11)	3.61 ± 2.99	3.82 ± 2.99	*p* = 0.074
Magnification scores (items 6, 7, and 13)	2.32 ± 2.23	2.07 ± 2.36*	*p* = 0.046
Helplessness scores (items 1, 2, 3, 4, 5, and 12)	3.36 ± 3.49	3.82 ± 3.89	*p* = 0.35

### Short-Form McGill Pain Questionnaire

Total scores, sensory scores, and VAS scores of the SF-MPQ met the assumptions of normality. However, affective scores and PPI scores of the SF-MPQ failed to meet normality assumptions, and therefore, for these, Friedman Test was used to compare pre-post-conditions, while Mann Whitney *U* test was used to analyze the sex effect. Statistical analysis of total scores showed a significant main time effect for the entire group; [*F*(1,26) = 5.57, *p* = 0.026]. Sensory scores also showed a significant main time effect for the entire group; [*F*(1,26) = 5.22, *p* = 0.031], and VAS scores revealed significant main time effect for the entire group; [*F*(1,26 ) = 11.77, *p* = 0.002]. Total scores, sensory scores, and VAS scores were significantly higher in the post-fatigue condition compared to the pre-fatigue condition. Statistical analysis using Friedman’s test for affective scores and Present Pain Intensity (PPI) scores showed no significant results. There were no significant differences between men and women for any of the SF-MPQ scores.

### Pain catastrophizing scale

All PCS data failed the Shapiro-Wilk test of normality, and therefore, non-parametric tests were used for all PCS statistical analysis. Only magnification scores showed a significant time difference for the entire group [*χ*^2^ (1) = 4, *p* = 0.046] with post-fatigue scores (*M* = 2.07, SD = 2.36) being lower than pre-fatigue scores (*M* = 2.32, SD = 2.23). When assessed according to sex using the Mann Whitney *U* test, there were no significant findings.

#### Relationships between questionnaires and pressure pain thresholds

**Fig. 1:**
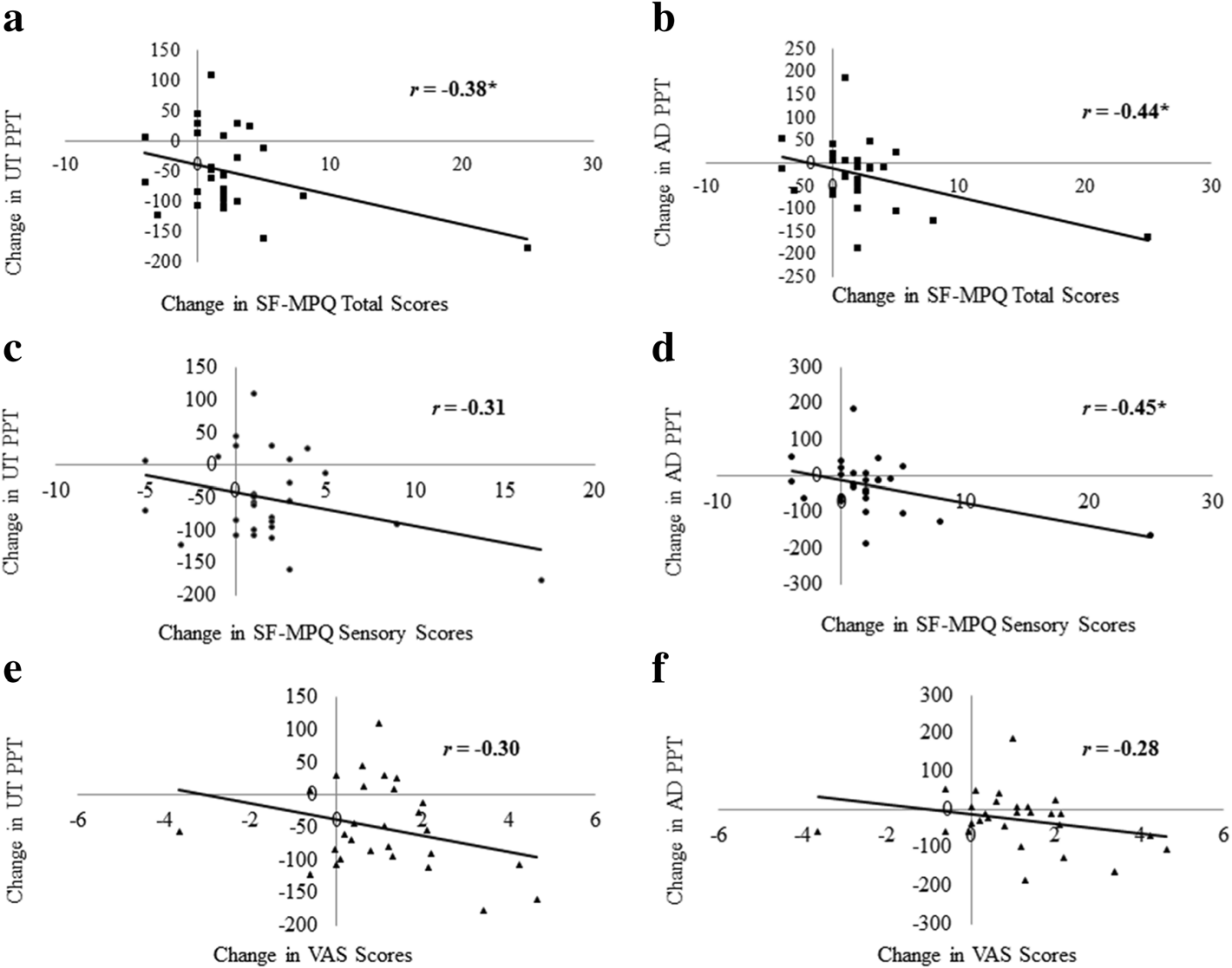
**a**–**f** Relationships between the changes in PPT and the changes SF-MPQ scores for the entire group (*statistically significant at *p* < 0.05)

Correlations between pre- and post-fatigue changes in total scores, sensory scores, and VAS scores of the SF-MPQ and changes in PPT for AD and UT muscle sites revealed three significant relationships (Fig. 1). There was a negative correlation between the change in UT PPT and the change in total scores of SF-MPQ [*r* = − 0.38, *p* = 0.047] with “poor to fair” strength. In other words, people who showed the largest increases in total scores of the SF-MPQ were also the ones who had the largest decreases in UT PPT. Negative correlations with “poor to fair” strength were observed between the change in AD PPT and the change in total scores of SF-MPQ [*r* = − 0.44, *p* = 0.019] and between the change in AD PPT and change in sensory scores of SF-MPQ [*r* = − 0.45, *p* = 0.017]. That is to say, those with the largest decrease in AD PPT also had the largest increases in total scores and sensory scores of the SF-MPQ. When each sex was looked at separately, there were no significant correlations seen for neither men nor women. No significant correlations were seen between changes in magnification scores of PCS and changes in UT PPT [*r*_*s*_ = − 0.13, *p* = 0.497] and AD PPT [*r*_*s*_ = − 0.07, *p* = 0.716] measures.

#### Correlations between pain measures and time to Borg-8

No significant relationships were found for any group between time to Borg-8 and pre-to post-changes in UT PPT (Table 3). However, only men showed a significant positive correlation between time to Borg-8 and change in AD PPT [*r* = 0.66, *p* = 0.01] with “moderate to good” strength, whereas there was no relationship between time to Borg-8 and change in AD PPT in women. In other words, men who performed the task for longer times also saw the largest increases in AD PPT from the pre- to post-conditions.

**Table 3:** Relationships between PPT and PCS pain measures and time to Borg-8 (*statistically significant at *p* < 0.05; **statistically significant at *p* < 0.01)

Change in PPT and time to Borg-8			
		Pearson’s correlations	Significance
UT	Entire group	0.27	*p* = 0.170
	Men	0.52	*p* = 0.058
	Women	− 0.06	*p* = 0.84
AD	Entire group	0.32	*p* = 0.10
	Men	0.66*	*p* = 0.01
	Women	0.15	*p* = 0.6
Pre-fatigue PCS Scores and time to Borg-8			
		Spearman’s rho	Significance
Total (all 13 items)	Entire group	− 0.41*	*p* = 0.032
	Men	− 0.17	*p* = 0.57
	Women	− 0.60*	*p* = 0.025
Magnification (items 6, 7, and 13)	Entire group	− 0.31	*p* = 0.114
	Men	− 0.33	*p* = 0.244
	Women	− 0.44	*p* = 0.119
Helplessness (items 1, 2, 3, 4, 5, and 12)	Entire group	− 0.50**	*p* = 0.007
	Men	− 0.13	*p* = 0.657
	Women	− 0.54*	*p* = 0.047
Rumination (items 8, 9, 10, and 11)	Entire group	− 0.29	*p* = 0.141
	Men	− 0.06	*p* = 0.841
	Women	− 0.46	*p* = 0.102

**Fig. 2:**
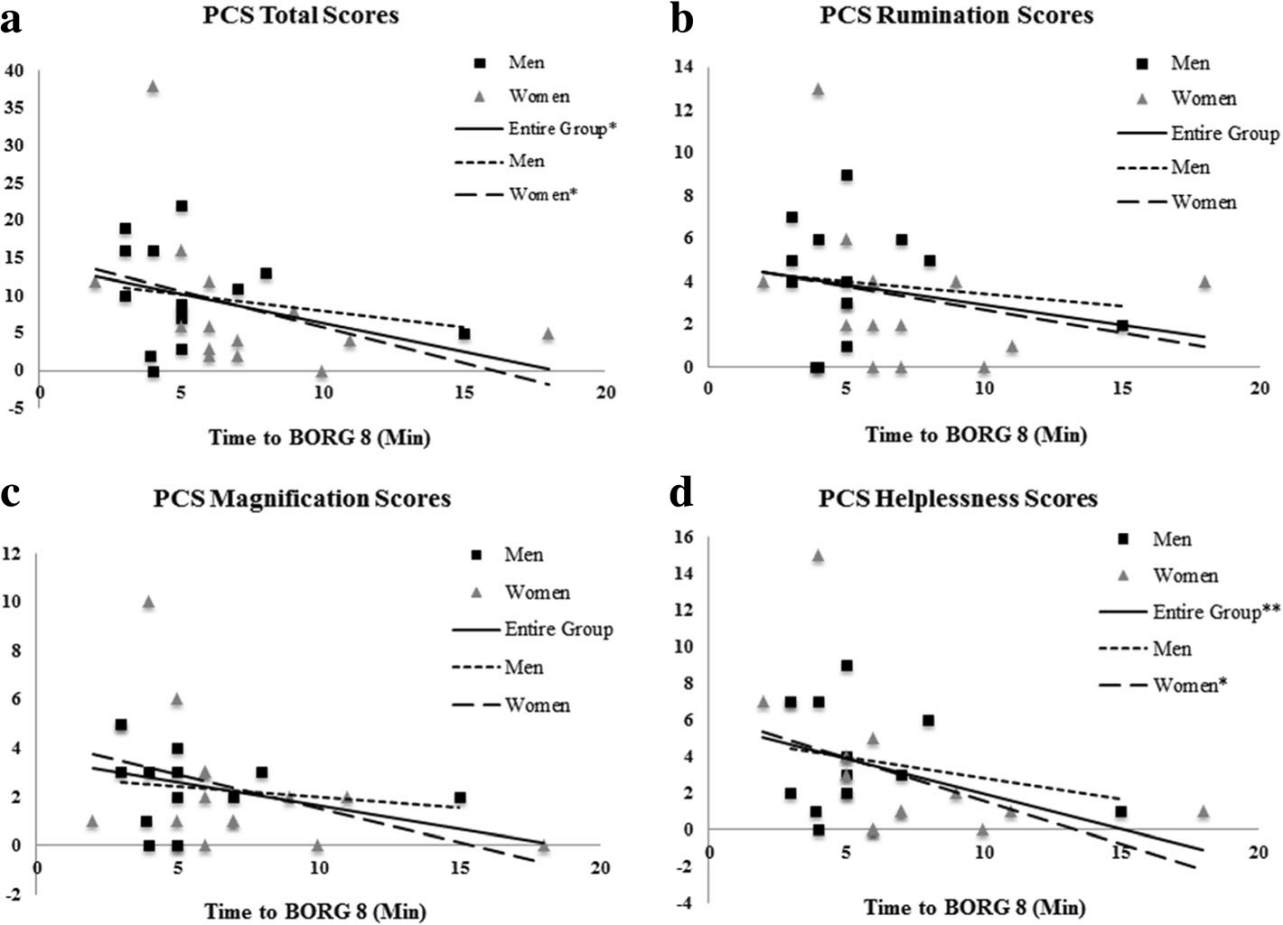
**a**–**d** Spearman’s rho correlations between time to Borg-8 and pre-fatigue PCS measures (*statistically significant at *p* < 0.05; **statistically significant at *p* < 0.01)

The entire group showed significant negative correlations between time to Borg-8 and both PCS total scores [*r*_*s*_ = − 0.41, *p* = 0.032] and PCS helplessness scores [*r*_*s*_ = − 0.50, *p* = 0.007], falling in the “poor to fair” strength range (Fig. 2). In other words, participants who had higher PCS scores performed the task for the shortest amount of time. However, when each sex was evaluated separately, only women showed significant negative correlations with “moderate to good” strength between time to Borg-8 and both PCS total scores [*r*_*s*_ = − 0.60, *p* = 0.025] and PCS helplessness scores [*r*_*s*_ = − 0.54, *p* = 0.047], whereas there were no significant relationships between time to Borg-8 and any of the PCS measures in the pre-fatigue condition for men.

## Discussion

In this study, the time to fatigue did not differ between men and women and is comparable to those in other studies using a similar fatiguing task [11]. Both men and women showed a linear progression of RPE scores throughout the duration of the experimental task, and all participants ended up reporting Borg 8/10 or higher while performing the task.

### Effects of the fatiguing task on PPT

Our results show that a low-load shoulder fatiguing task influences pressure pain thresholds (PPT), with upper trapezius PPT values significantly decreasing with fatigue. The literature on the effects of exercise on PPT is currently equivocal [8], with studies showing increases, no effect, or decreases in PPT. However, results from various studies point to important impacts of the kind of task (eccentric, isotonic, or isometric), and muscle(s) investigated to explain variable results. As such, the studies investigating isometric tasks (similar to what the shoulder experienced in the current study) were mainly conducted on lower limb and orofacial muscles (see [8] for a review), with one study conducted on a shoulder (infraspinatus) muscle [20], the majority showing increases in pain following isometric exercise. However, no studies have investigated PPT among multiple muscles of the neck/shoulder region following an upper limb, work-like task resembling the one used in the current study.

Our findings also demonstrate that anterior deltoid PPT levels of women were significantly lower than those of men, in line with the existing literature [1, 12, 13, 27]. A review by Rollman and Lautenbacher [29] has provided evidence supporting that there are sex differences in PPT, more so than in other pain modalities. Authors also suggest that mechanical pressure protocols are especially well suited to model the mechanisms of musculoskeletal disorders, and argue that women’s lower pressure pain threshold, in particular, may reflect an elevated sensitivity to deep tissue pain that may be the cause of musculoskeletal pain. Thus, we can hypothesize that the lower PPT levels in the anterior deltoid of women represent an increased risk of developing chronic musculoskeletal pain due to the likelihood of greater overall pain sensitivity, regardless whether there is muscle fatigue. Finally, our observations of sex differences in AD PPT only, and, conversely, fatigue effects in UT PPT only, may reflect differences in muscle fiber characteristics, in pain pathways, and/or in how our experimental task affected each muscle. Indeed, we have previously shown that men and women engage both muscles differently during the performance of this experimental task, both before and after fatigue (see [6] for a review), which could have repercussions on their mechanical pain sensitivity, although this should be verified with further studies of larger sample sizes.

Furthermore, only men indicated changes in anterior deltoid PPT levels that were positively related to the amount of time the task was performed. Thus, men who performed the task for the longest durations also had the largest increases in PPT following the neck/shoulder fatiguing task. Similarly, electromyography (EMG) analyses published previously showed that only men display a relationship between perceived exertion and AD EMG [25]. Taken together, this could reflect that men are better able to modify their sensorimotor patterns in response to fatigue elicited during such shoulder height tasks, which could help men develop injury-preventative strategies earlier and thus more effectively, although this interpretation is speculative.

### Effects of fatigue on verbally described pain and pain intensity

Our results show that the task increased verbally described pain from the SF-MPQ and VAS intensity ratings of musculoskeletal pain. Hollander et al. [15] used the SF-MPQ to analyze descriptive pain perception during isometric, eccentric, and concentric contractions and found that scores increased over time, with a greater increase seen in the sensory dimension of pain. This is consistent with present results, as total scores and sensory scores also showed increases. Furthermore, our results agree with previous literature that has found increases in pain intensity ratings during and immediately after various types of fatiguing contractions for healthy participants [8].

In our study, the increases in verbally described pain and pain intensity complement the decreases in upper trapezius PPT, as together these findings indicate an increase in physical and verbal sensitivity to muscle pain with fatigue. To further support this, greater increases in SF-MPQ total scores were related to greater decreases in upper trapezius PPT. Moreover, this same relationship was also observed between changes in anterior deltoid PPT and in the SF-MPQ, both in terms of SF-MPQ total scores and sensory scores. Thus, although only the upper trapezius shows a significant change with the experimental task, pain perceptions at both the upper trapezius and anterior deltoid contribute to the verbally described sensation. Given the significant relationships between changes in anterior deltoid PPT and in both general and sensory SF-MPQ, and the importance of the anterior deltoid in shoulder height tasks, the SF-MPQ may be viewed as a complementary, adjunct tool to help detect signs of fatigue-related neck/shoulder pain. This might be especially pertinent to women since, as hypothesized above, women may not be as effective at using fatigue-related changes in a proactive way as men may be able to.

While acute pain sensitivity increases (as measured from increases in UT PPT and in both pain intensity and verbally described pain), general pain magnification for the neck/shoulder area, as measured by the PCS, decreases. As magnification is considered a primary appraisal for the threat of a painful situation [31], these decreases indicate that when people experience acute muscle pain in the presence of fatigue, less of an emphasis is placed on the importance of the painful threat than when in a non-fatigued state. This could lead us to believe that when men and women consider their overall thoughts on pain in the neck/shoulder area in a fatigued state, they are less likely to exaggerate general neck/shoulder pain. The lack of correlations seen between changes in PCS magnification scores and changes in PPT measures is not surprising, as they refer to two different kinds of pain. Indeed, the previous literature has shown relationships between PCS and some types of experimentally induced pain, such as cold pressor pain in a non-fatigued state [31], and not specifically PPT. Furthermore, no sex differences were observed for any of the SF-MPQ scores or PCS scores. While a sex comparison has, to our knowledge, never been conducted with the SF-MPQ, this is in contrast with the current literature on pain catastrophizing, where women have been shown to engage in greater levels of catastrophizing following the application of a noxious stimulus compared to men [1, 12, 31]. However, it has been shown that women specifically engage in rumination and helplessness dimensions of catastrophizing, while sex differences are not seen in magnification dimension of catastrophizing [31]. Therefore, these findings could be related to the fact that only a significant time difference was observed in the magnification scores.

Nevertheless, correlational analyses indicated that baseline pain catastrophizing levels were related to the amount of time one could perform the neck/shoulder fatiguing task. When looking at the entire group, higher baseline PCS scores were linked with lower time to Borg-8. What’s more, when each sex was evaluated separately, the relationship between time to Borg-8 and both PCS total scores and PCS helplessness scores only remained for women, and not men. These results suggest that women who actively engage in more pain catastrophizing, specifically helplessness in the presence of pain, are less able to cope with the pain sensation or even the thought of the pain sensation. This fits within the fear-avoidance model, which suggests that, following acute injury, engaging in pain catastrophizing leads to fear of pain, and possibly pain anxiety, and subsequently promotes injury avoidance behavior [33]. Moreover, these findings are more consistent with the current literature indicating that men and women differ in their levels of and responses to pain catastrophizing [1, 9].

### Limitations

Results from this study are limited to neck/shoulder pain and exertion perceptions of young, healthy adult men and women for a low-load, work-like fatiguing task of a few minutes duration. There are several factors that can influence pain expression and the pain response, such as hormonal levels, socio-cultural influences, and previous pain experience [12], some of which may not be reflected in this study. Also, there are limitations associated to our implementation of the PPT method and to the use of the PCS to specifically assess attitudes towards neck/shoulder pain. Finally, the small sample size likely affected the power of some comparisons.

### Conclusions

Our results show that low-load shoulder height work increases pain sensitivity in shoulder stabilizers (upper trapezius), but not mobilizers (anterior deltoid). In addition, our results suggest that the SF-MPQ may be a good tool to supplement physical feedback provided during neck/shoulder fatiguing tasks in developing proactive, injury-preventative strategies, especially for women. These findings could be relevant to our understanding of the sex-specific pain and injury mechanisms. In turn, a better understanding may lead to better rehabilitation and injury prevention approaches.

## Data Availability

Please contact the corresponding author for data requests

## Abbreviations

AD: Anterior deltoid
ANOVA: Analysis of variance
BIC: Biceps Brachii
Borg-8: Rating of 8/10 on the Borg CR10 scale for neck/shoulder RPE
CR10: Category ratio 10
EMG: Electromyography
MSD: Musculoskeletal disorder
PCS: Pain Catastrophizing Scale
PPI: Present Pain Intensity
PPT: Pressure pain threshold
RPE: Rating of perceived exertion
SF-MPQ: McGill Pain Questionnaire (Short Form)
UT: Upper trapezius
VAS: Visual analog scale
WMSD: Work-related musculoskeletal disorder
